# Functioning and mechanisms of PTMs in renal diseases

**DOI:** 10.3389/fphar.2023.1238706

**Published:** 2023-11-21

**Authors:** Zhenzhen Liu, Jian Yang, Minghui Du, Wei Xin

**Affiliations:** ^1^ Medical Science and Technology Innovation Center, Shandong First Medical University and Shandong Academy of Medical Sciences, Jinan, China; ^2^ Biomedical Science College, Shandong First Medical University, Jinan, China; ^3^ Shandong Provincial Hospital Affiliated to Shandong First Medical University, Jinan, China

**Keywords:** post-translational modification, diabetic kidney disease, acute kidney injury, histone modifications, renal disease

## Abstract

Post-translational modifications (PTMs) are crucial epigenetic mechanisms that regulate various cellular biological processes. The use of mass spectrometry (MS)-proteomics has led to the discovery of numerous novel types of protein PTMs, such as acetylation, crotonylation, 2-hydroxyisobutyrylation, β-hydroxybutyrylation, protein propionylation and butyrylation, succinylation, malonylation, lactylation, and histone methylation. In this review, we specifically highlight the molecular mechanisms and roles of various histone and some non-histone PTMs in renal diseases, including diabetic kidney disease. PTMs exhibit diverse effects on renal diseases, which can be either protective or detrimental, depending on the specific type of protein PTMs and their respective targets. Different PTMs activate various signaling pathways in diverse renal pathological conditions, which could provide novel insights for studying epigenetic mechanisms and developing potential therapeutic strategies for renal diseases.

## Introduction

The kidney, an essential organ in the human body, plays a vital role in maintaining physiological functions, such as the elimination of excess water via the urine, regulating ion balance through filtration, reabsorption, and secretion, and serving as the site of degradation for some endocrine hormones and the target organ of extrarenal hormones. These functions are critical for maintaining the body’s internal environment and normal metabolism (Koye et al., 2018). However, renal diseases are becoming increasingly a severe health issue, with rising morbidity and mortality rates. Epidemiological analysis indicates that renal disease currently affects more than 750 million people globally, making it the most significant public health concern worldwide (Crews et al., 2019). Therefore, urgent attention and appropriate preventive measures are crucial to combat this challenge (Arici, 2021).

Diabetic kidney disease (DKD), acute kidney injury (AKI), renal fibrosis, and polycystic kidney disease (PKD), presents a significant threat to global public health. Of these, CKD is considered a major concern, leading to increased healthcare costs worldwide (Levin, 2018). The prevalence of CKD was reported to be 9.1% (697.5 million cases) globally in 2017, resulting in 1.2 million deaths and ranking it as the 12th leading cause of death worldwide (Carney, 2020). DKD, a significant contributor to CKD, is one of the most serious complications of diabetes, leading to glomerulosclerosis and end-stage renal disease (ESRD)(Dronavalli et al., 2008). Approximately 50% of patients with diabetes develop DKD, and consequently ESRD, 20 years after diabetes onset (Packham et al., 2012). Notably, approximately 40%–45% and 30% of patients with type 1 and type 2 diabetes develop DKD, respectively (Oltean et al., 2017; Dong et al., 2021). AKI is another crucial factor contributing to CKD, with approximately 13 million cases worldwide each year, causing 1.7 million deaths, prolonged hospitalization, and increased healthcare costs (Mehta et al., 2015; Mehta et al., 2016). Despite the increasing burden of renal diseases on global health, there is still no satisfactory treatment (Mehta et al., 2016). Therefore, understanding the mechanisms of post-translational modifications (PTMs) in renal diseases is essential for identifying novel targets for therapy development. Additionally, epigenetic involvement has been suggested in the pathogenesis of renal disease in many cases, providing further insights into the molecular basis of renal diseases.

Epigenetics is a process of heritable gene expression changes without any changes in nucleotide sequence. This process involves various mechanisms, including DNA methylation, histone modifications, and microRNAs, which can lead to heritable phenotypic changes without altering the DNA sequence (Zhang et al., 2020a). The study of epigenetic mechanisms involved in gene expression regulation has emerged as an effective method, providing new insights for the clinical treatment of different diseases. Histone modifications are among the crucial epigenetic mechanisms that have gained significant attention in renal diseases recently. Nucleosomes, which are the basic building blocks of chromatin, consist of double-stranded DNA and four basic histones, including H2A×2, H2B×2, H3×2, and H4×2, that form an octameric core histone. These histones are rich in lysine and arginine residues, making them easily modifiable (Kouzarides, 2007; Li et al., 2019). The N and C termini of histones can undergo PTMs, such as acetylation, methylation, SUMOylation, and ubiquitination (Strahl and Allis, 2000). These modifications can alter the charge and structure of histone tails bound to DNA, modify the chromatin state, and subsequently positively or negatively regulate gene expression (Chen et al., 2014). Histone modifications have been found to play a crucial role in the pathogenesis of various diseases (Tang and Zhuang, 2019; Bajbouj et al., 2021; Wang et al., 2022a).

**FIGURE 1:**
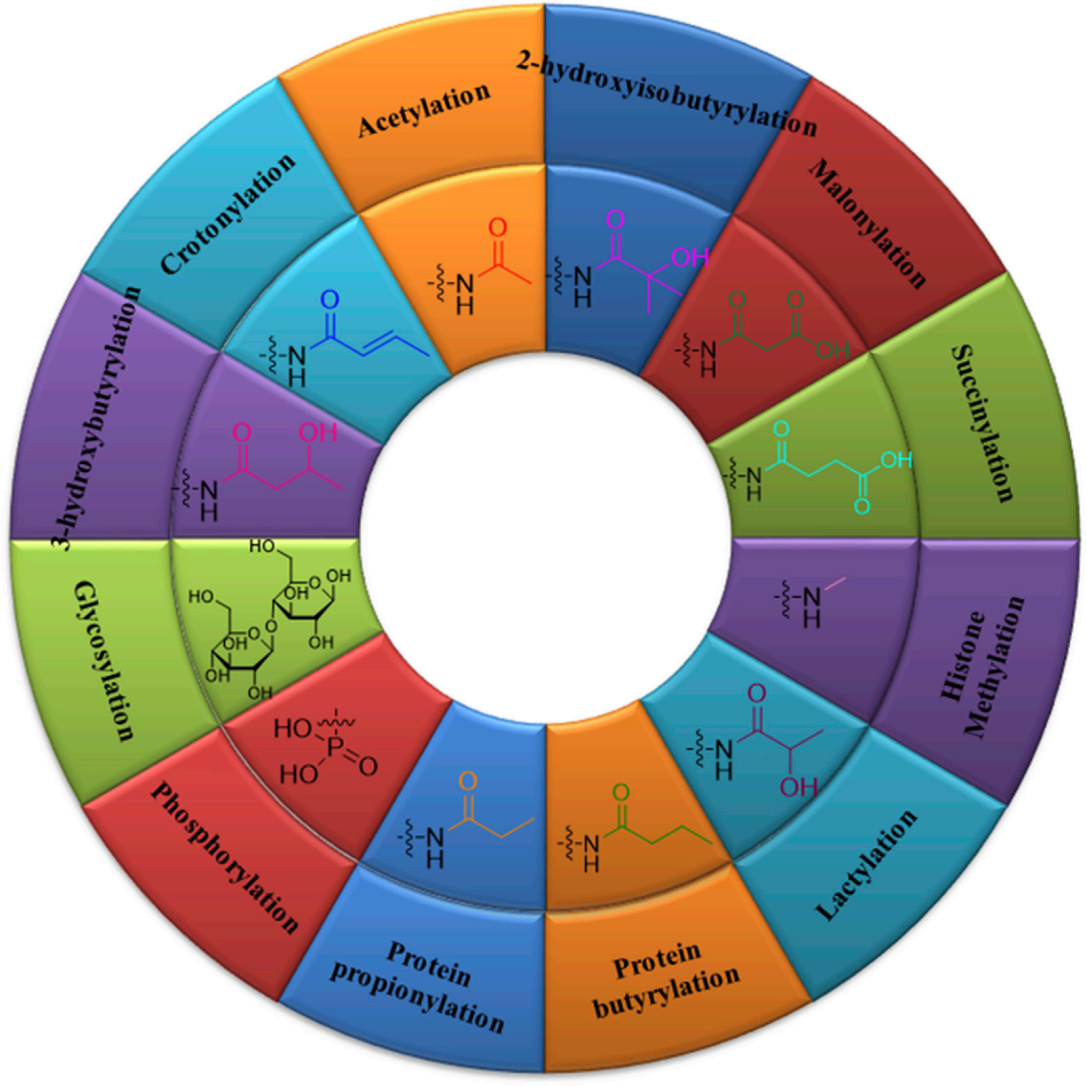
Types and structures of post-translational modifications: acetylation, glycosylation, phosphorylation, crotonylation, 2-hydroxyisobutyrylation, β-hydroxybutyrylation, protein propionylation and butyrylation, succinylation, malonylation, lactylation, and histone methylation.

**FIGURE 2:**
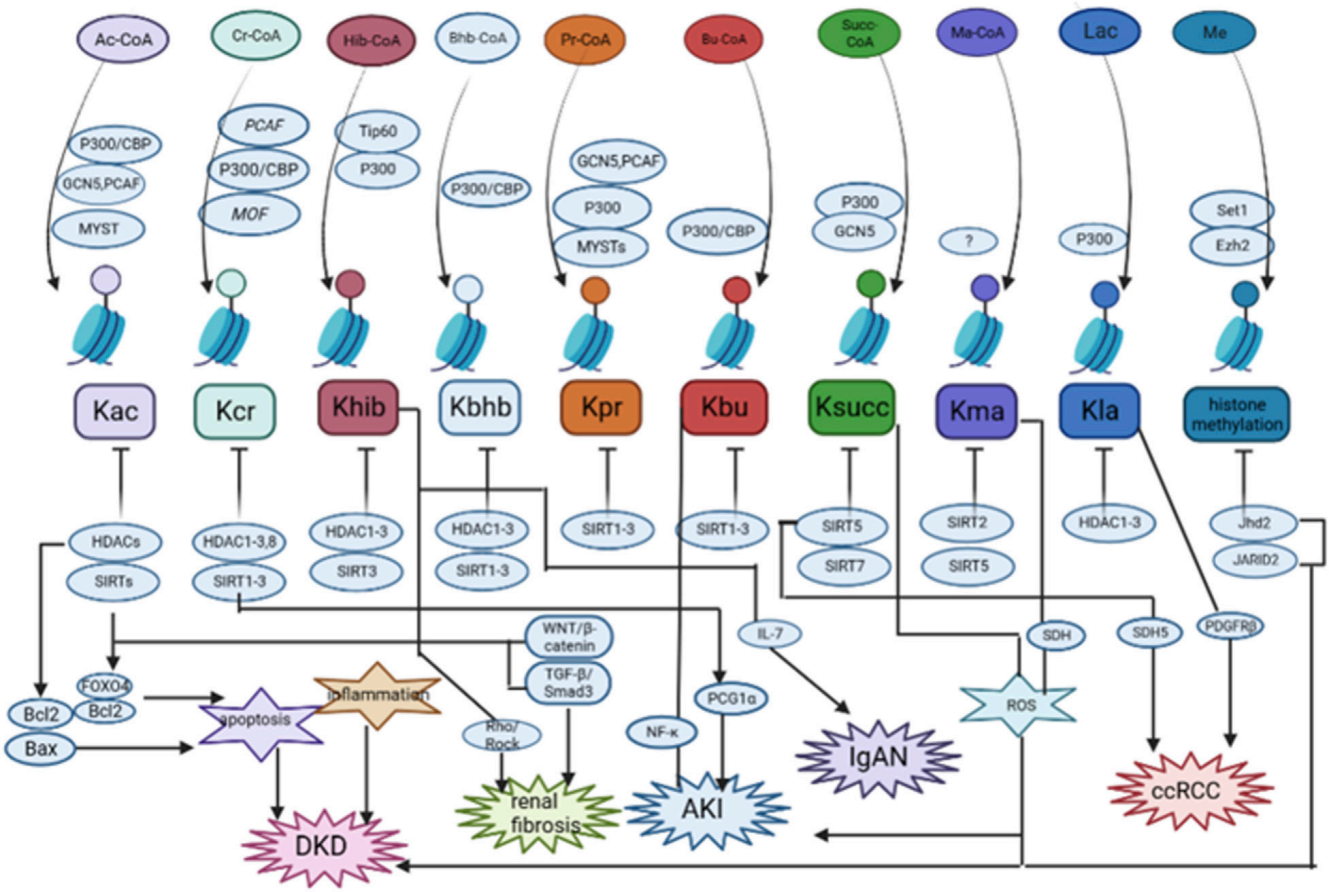
The summary of the role and mechanisms of histone post-translational modifications (HPTMs) in renal diseases. Most HPTMs originate from acyl-CoA. It has been verified that HPTMs participate in the regulation of apoptosis, inflammation, Wnt/β-catenin signaling, TGF-β signaling, and so on in renal diseases. DKD: diabetic kidney disease; AKI: acute kidney injury; IgAN: IgA nephropathy; ccRCC: clear cell renal cell carcinoma. Histone methylation was only studied for H3K4 and H3K27 methylation.

The transcription of numerous genes and activation of various signaling pathways involved in the pathogenesis of renal diseases are associated with histone PTMs (HPTMs) (Lin et al., 2014a; Huang et al., 2014; Huang et al., 2021a). Therefore, targeting these modifications could be a promising strategy to safeguard the kidney and promote renal function repair and recovery. This review primarily focuses on the latest research developments on HPTMs in CKD, DKD, AKI, renal fibrosis, and other renal diseases. It explores the role of histone modifications in the regulation, modification, and significance of these diseases (Figures 1, 2).

## PTMs in renal diseases

PTMs are covalent changes in proteins or peptides that increase the functional diversity of the proteome through the covalent addition of functional groups or proteins, proteolytic cleavage of regulatory subunits, or degradation of the entire protein. This increases the complexity of protein regulation by affecting active states, subcellular localization, turnover, and interactions with other cellular molecules (Mann and Jensen, 2003). With the critical role of functional proteomics, an increasing number of protein PTM types have been found to be associated with the development and progression of various renal diseases (Table 1). Lundby et al. reported that among the tissues examined, the kidney contained the most acetylated proteins, and the largest acetylated proteome was localized to the plasma membrane of the kidney and was involved in ion transport. Regulation of the balance between acetylation and deacetylation is critical in maintaining renal function (Hyndman and Knepper, 2017). Glycosylation promotes the proliferation and apoptosis of tubular epithelial cells and is involved in the progression of renal diseases (Xu et al., 2023). Tyrosine phosphoproteins are concentrated in glomeruli to play a critical role in their signaling (Denhez and Geraldes, 2017). Lysine crotonylation, 2-hydroxyisobutyrylation, 3-hydroxybutyrylation, protein propionylation and butyrylation, succinylation, malonylation, lactylation, and other novel short-chain lysine acylation reactions originating from short-chain fatty acids (SFCA) have received increasing attention. For example, butyrate and propionate can prevent AKI induced by ischemia-reperfusion in mice as well as diabetic nephropathy (Andrade-Oliveira et al., 2015; Huang et al., 2020). In sepsis-induced acute kidney injury (SAKI), a lactate-mediated increase in Fis1 lactate levels promotes excessive mitochondrial division, which subsequently leads to excessive mitochondrial reactive oxygen species (MtROS) production and mitochondrial apoptosis, providing a new perspective on novel targets for AKI treatment (An et al., 2023). PTMs are involved in the development of kidney disease, and the mechanism may be related to inflammatory responses, oxidative stress, mitochondrial function, autophagy and apoptosis of kidney cells, and other physiological processes to protect the kidneys (Table 2). In this part, we focus on the research progress of histone and some non-histone post-translational repair in kidney diseases, especially DKD.

**TABLE 1:** Summary of types of post-translational modifications in renal diseases.

Post-translational modifications	Writers	Readers	Erasers	Involved renal diseases	References
**Acetylation**	GCN5, PCAF P300/CBP MYST	Bromodomaindou DPF domain YEATS domain	HDACs: Class I(HDAC1-3,8); Class II (HDAC4-7,9,10); Class IV (HDAC-11) SIRT1-7	renal-proliferation AKI DKD renal fibrosis ccRCC	Villanueva et al. (2006), Wang et al. (2014), Cai et al. (2020), Cui et al. (2022b), Zhang and Cao, 2022
**Glycosylation**	GTs (GT-A,GT-B,GT-C)	-	GHs	DKD IgAN renal fibrosis	Huang et al. (2016), Cannizzaro et al. (2017), Morais et al. (2023), Xu et al. (2023)
**Phosphorylation**	STKs,TKs,DSKs(AGC,CAMK,CK1,CMGC,MAPKs,GSK3,CDK2,STE,TK,TKL)	SLiMs	PTPs,PPPs(PP1, PP2A, PP2B, PP4–PP7)PPMs),DUSPs	DKD AKI renal fibrosis	Tikoo et al. (2001), Ardito et al. (2017), Alghamdi et al. (2018), Kliche and Ivarsson (2022), Lin et al. (2023)
**Crotonylation**	P300/CBP, PCAF, MOF	DPF domain YEATS domain CDYL	HDAC1-3,8 SIRT1-3	AKI	Martinez-Moreno et al. (2020a)
**2-hydroxyisobutyrylation**	P300 Tip60	_	HDAC1-3 SIRT3	renal-proliferation AKI IgAN renal fibrosis ESRD	Huang et al. (2021b), Perico et al. (2021), Zheng et al. (2021)
**β-hydroxybutyrylation**	P300/CBP	_	HDAC1-3 SIRT1-3	DKD PKD	Luo et al. (2020a)
**Propionylation**	P300 GCN5/PCAF MYSTs	Bromodomaindou YEATS domain	SIRT1-3	ESRD	Meyer et al. (2020a)
**Butyrylation**	P300/CBP	Bromodomaindou YEATS domain	SIRT1-3	DKD	Luo et al. (2020a)
**Succinylation**	P300 GCN5	YEATS domain	SIRT5 SIRT7	ccRCC	Meyer et al. (2020a)
**Malonylation**	_	_	SIRT2 SIRT5	_	_
**Lactylation**	P300	_	HDAC1-3	ccRCC	Yang et al. (2022)
**Histone Methylation (H3K4, H3K27)**	Set1 Ezh2	PHD domain Chromo domain	Jhd2 JARID2	DKD	Chen et al. (2019), Cao et al. (2021)

**TABLE 2:** PTM mechanisms in renal diseases.

PTM	Target	Pathway (target)	Biologic effects	Renal diseases	Reference
**Kac**	SIRT1	PGC-1α	reduced oxidative stress	DKD	Hong et al. (2018)
	SIRT1	FOXO4/BCL2	reduced apoptosis	DKD	Wakino et al. (2015), Chen et al. (2020)
	SIRT1	P53/AMPK	enhanced autophagy and mute inflammation and apoptosis	DKD	Dong et al. (2021)
	SIRT1	TGF-β/Smad3	reduced extracellular matrix protein expression	CKD	Huang et al. (2014)
	SIRT1	P53/NF-κB	Enhanced inflammation	CKD	Fu et al. (2022)
	SIRT1	P53	alleviated oxidative stress and reduced apoptosis	AKI	Li et al. (2019)
	SIRT1	HMGB1	mute inflammation	SA-AKI	Wei et al. (2019)
	SIRT1	PGC-1α	activated mitochondrial biogenesis and respiration via oxidative phosphorylation	AKI	Funk and Schnellmann (2013)
	SIRT3	SOD2	reduced oxidative stress	DKD	Locatelli et al. (2020)
	SIRT3	TGF-β/Smad3	led endothelial-to-mesenchymal transition (EndMT)	DKD	Sriyastava et al. (2021)
	SIRT6	Notch	enhanced autophagy	DKD	Liu et al. (2017)
	SIRT6	WNT/β-catenin	decreased fibrotic gene expression and suppressed renal fibrosis	Renal fibrosis	Cai et al. (2020), Jin et al. (2022)
	SIRT6	TGF-β/Smad3	Inhibited nuclear accumulation and transcriptional activity	DKD	Wang et al. (2021)
	HDAC4	miR-29a/HDAC3/H3K9Ac	reduced podocyte apoptosis, glomerular fibrosis, inflammation, and renal dysfunction	DKD	Lin et al. (2014a)
	HDAC3	p27Kip1	mute cell proliferation	Kidney development	Zhang et al. (2021)
	HDAC4	BAX/BCL2	reduced apoptosis	DKD	Shi et al. (2020)
	HDAC5	BMP7	promoted regeneration of the injured kidney	I/R kidney Injury	Marumo et al. (2008)
	GCN5L1	TFAM	led mitochondrial dysfunction	AKI	Lv et al. (2022)
	GCN5L1	MnSOD	relieved oxidative stress-induced renal inflammation and fibrosis	DKD	Lv et al. (2021)
	p300	SNIP/TGF-β/SMAD4	Increased histone acetylation and the activation of cell movement related genes	ccRCC	Cui et al. (2022b)
**Glycosylation**	ENTPD5	N-glycosylation	promoted proliferation or apoptosis of tubular epithelial cells	DKD	Xu et al. (2023)
	IgA1	O-glycosylation	resulted in the formation of pathogenic immune complexes and induced glomerular injury	IgAN	Berthoux et al. (2012)
**Phosphorylation**	SIRT3	MAPK/NF-κB	regulated mitochondrial oxidative capacity and expression of antioxidant genes	proteinuric kidney disease	Koyama et al. (2011)
	SIRT6	AMPK	mute apoptosis	DKD	Fan et al. (2019)
	SIRT6	NF-κB	reduced inflammation	renal fibrosis	You et al. (2019)
		ATM/γH2AX	regulated mitochondrial injury and tubular cell apoptosis by DDR	AKI	Ma et al. (2014)
	H3Ser10 phosphorylation	p38 MAPK/MSK1/2	facilitated glomerular endothelial activation	DKD	Alghamdi et al. (2018)
	FADD	TLR4/myD88/NF-Κb, mTOR, TGF-β/Smad	indicated EMT	renal fibrosis	Lin et al. (2023)
	MAP4	p38/MAPK -MAP4	prevented the dediferentiation and apoptosis	DKD	Li et al. (2022)
	Cdk5	MEKK1/JNK	reduced podocyte apoptosis	DKD	Zhang et al. (2017)
**Kcr**	SIRT3	PGC-1α	mute inflammation	AKI	Martinez-Moreno et al. (2020b)
**Ksucc**	SIRT5	SDHA	promoted cell proliferation	ccRCC	Meyer et al. (2020a)
**Kma**	SIRT5	aldolase A and B	Involved in glycolysis and peroxisomal fatty acid oxidation	DKD	Baek et al. (2023)
**Histone methylation**	UTX	Jagge-1	facilitated inflammation and DNA damage	DKD	Chen et al. (2019)

## Acetylation

Histone acetylation (Kac), which involves the transfer of acetyl groups to lysine residues, has garnered attention as a prominent type of histone modification in renal pathogenesis. It alters the charge of histone, leading to chromatin relaxation, and facilitates the binding of transcription factors to promoters, thus promoting gene expression. The dynamic balance of histone acetylation and deacetylation is regulated by two opposing enzymes, histone acetyltransferase (HAT) and histone deacetylase (HDAC), respectively (Chen et al., 2014). HATs can be grouped into three major families: GCN5, p300, and MYST. Similarly, HDACs are divided into four classes: classes I (HDAC-1, 2, 3, and 8), II (HDAC-4, 5, 6, 7, 9, and 10), III sirtuin family (SIRT1-SIRT7), and IV (HDAC-11)(Bhaumik et al., 2007). HATs and HDACs also regulate the acetylation and deacetylation of non-histone proteins and are also referred to as lysine acetyltransferases and lysine deacetylases, respectively (Narita et al., 2019). Acetylation of numerous genes involved in kidney function can be regulated by these enzymes.

Acetylation is a well-known histone modification type that has been linked to diabetes pathogenesis. HDAC has been identified as a critical player in DKD, with upregulation of HDAC-2, 4, and 5 detected in the kidneys of diabetic rats, db/db mice, and renal biopsy samples of patients with diabetes (Wang et al., 2014). In a 2020 study by Shi et al., 2020, HDAC-4 was found to be involved in podocyte apoptosis in DKD. Stimulation of podocytes with FK506, a CaN inhibitor, after overexpression of HDAC-4, decreased CaN and reduced Bax expression while increasing Bcl-2 expression, ultimately attenuating podocyte apoptosis caused by HDAC-4 overexpression (Shi et al., 2020). Furthermore, renin instability has been shown to decrease podocyte survival and accelerate diabetes-induced renal damage, while HDAC-4 signaling is involved in renin stability in the progression of diabetes-induced podocyte injury. Inhibition of HDAC-4 has been demonstrated to restore H3K9ac enrichment at the miR-29a promoter region and increase miR-29a transcription in the high glucose-stimulated podocyte state. Inhibition of HDAC-4 also restores the acetylation status of nephrin, thereby improving diabetes-induced podocyte injury and renal dysfunction. Additionally, miR-29a overexpression significantly reduces HDAC4 levels, highlighting the epigenetic involvement in the pathological process of podocytes in DKD (Lin et al., 2014a).

Mammalian SIRT1, a conserved nicotinamide adenine dinucleotide + -dependent protein deacetylase of the sirtuin family, is expressed widely in mammalian cells (Afshar and Murnane, 1999) and plays a crucial role in various biological processes, including aging (Xu et al., 2020), inflammation (Nakamura et al., 2017), cancer (Zhang et al., 2015), metabolism (Hong et al., 2015), and neurodegenerative diseases (Dobbin et al., 2013). SIRT1, along with other sirtuins, also contributes significantly to the development of renal disease (Hao and Haase, 2010). In particular, SIRT1 expression in podocytes and glomerular cells of human diabetic kidneys and the overall reduction of SIRT1 in db/db mice have been shown to accelerate DKD progression, whereas tubular SIRT1 expression mitigates diabetic glomerular injury reduction and elevates SIRT1 expression in glomerular podocytes, to attenuate DKD. Overexpression of SIRT1 in OVE diabetic mice has been found to reduce podocyte foot process effacement, glomerular basement membrane thickening, glomerular hypertrophy, and mesangial matrix expansion, suggesting that SIRT1 plays a role in and enhances the progression of DKD (Hong et al., 2018). SIRT1 also reduces apoptotic degeneration of podocytes and urinary protein by deacetylating FOXO4 and suppressing the expression of pro-apoptotic factor Bcl2. Similarly, reduced expression of SIRT1 in proximal tubules affects glomerular function (Hasegawa et al., 2013; Wakino et al., 2015). Additionally, inhibition of miR-150-5p promotes the interaction between SIRT1 and p53, reduces p53 acetylation in podocytes and renal tissue, and exerts a protective effect on the kidney in DKD mice (Dong et al., 2021). In the kidneys, the most extensively studied sirtuin is SIRT1, which, together with SIRT3, exerts cytoprotective effects by inhibiting apoptosis, inflammation, and fibrosis. This important metabolic sensor regulates ATP production and mitochondrial adaptive responses to stress. Yoshio et al. found that mitochondrial oxidative stress was caused by decreased activities of superoxide dismutase 2 (SOD2) and isocitrate dehydrogenase 2 (IDH2) in the kidneys of type 2 diabetic rats, which was associated with a decreased intracellular NAD +/NADH ratio and Sirt3 activity (Ogura et al., 2018). Further studies later found that inhibition of CD38 with apigenin in the kidneys of diabetic rats reduced tubular cell injury and proinflammatory gene expression by increasing the intracellular NAD+/NADH ratio and SIRT3-mediated mitochondrial antioxidant enzyme activity (Ogura et al., 2020). In 2020, Monica et al. found that Honokiol was able to activate SIRT3 signaling, and diabetic mice treated with Honokiol had increased SIRT3 activity, decreased SOD2 acetylation levels, and restored levels of NRF2, thus exerting a potential mechanism of its antioxidant activity to protect the kidneys (Locatelli et al., 2020). In addition, SIRT3 overexpression suppressed NF-κB-dependent transcriptional activity of inflammatory genes, decreased phosphorylation of ERK1/2 and p38, and decreased ROS levels, indicating a possible molecular mechanism of SIRT3-mediated antioxidant and anti-inflammatory effects in proximal tubular cells (Koyama et al., 2011).SIRT3 deficiency has been reported to promote mesenchymal transformation of tubular epithelial cells, thereby inducing fibrosis in DKD (Sriyastava et al., 2021). SIRT6 is also a potential therapeutic target for preventing and delaying DKD. Liu et al. found that Sirt6 expression was downregulated in renal biopsies from podocyte inury patients and correlated with the glomerular filtration rate. In patient and mouse models of DKD, reduction of SIRT6 levels leads to increased levels of H3K9ac in *Notch1* and *Notch4* promoters, thereby enhancing transcription of *Notch1* or *Notch4* genes. Activation of Notch signaling ultimately leads to podocyte injury by inducing inflammation, apoptosis, actin cytoskeleton disorganization, as well as inhibition of autophagy. Among target genes downstream of the Notch signaling pathway, *HES1* and *Snail* are mainly associated with proteinuria in renal diseases. Further, it was found that Snail and HES1 expression in podocytes was promoted in a high-glucose environment, and Sirt6 significantly inhibited their induction. Mitochondrial function is impaired under high-glucose conditions, and the transfection of plasmids overexpressing Sirt6 has been found to protect mitochondrial function and reduce oxidative stress by increasing AMPK phosphorylation (Liu et al., 2017; Fan et al., 2019; Yang et al., 2022a). At the same time, SIRT6 reduced the mRNA levels of inflammation-related factors IL-1β, IL-6, and TNF-α in podocytes (Liu et al., 2017). This evidence suggests that SIRT6 plays a protective role in high glucose-induced renal injury by reducing oxidative stress, mitochondrial damage, and inflammation, demonstrating Sirt6 as a potential therapeutic target in DKD (Yang et al., 2022a).

Previous research has established that SIRT1 can modify numerous transcription factors, including Smad3, a transcription factor that plays a significant role as a fibroblast mediator of transforming growth factor (TGF)-β, which is implicated in the development of CKD. The activation of SIRT1 has been shown to inhibit TGF-β/Smad3 signaling, thereby reducing Smad3 acetylation and improving renal fibrosis in 5/6 nephrectomized rats (Huang et al., 2014). Recent studies have identified that the activation of P53 inhibits the expression of SIRT1, resulting in increased p65 acetylation and nuclear factor (NF)-κB activation in the pathogenesis of CKD induced by repeated low-dose cisplatin treatment. This leads to the development of chronic kidney inflammation (Fu et al., 2022). Additionally, the SIRT1-p53 pathway plays a crucial role in cisplatin-induced premature renal failure and renal fibrosis induced by cisplatin treatment. Cisplatin triggers mitochondrial dysfunction and increased reactive oxygen species (ROS) production, which down-regulates the expression of SIRT1 and increases the level of acetylated p53, resulting in premature renal failure and, in turn, renal interstitial fibrosis (Li et al., 2019). Mitochondrial SIRT3 has been reported to play a critical role in the regulation of mitochondrial integrity and metabolism. Endothelial SIRT3 is an important anti-fibrotic molecule in diabetic kidneys. In diabetic mouse kidneys, endothelial cell (EC) SIRT3 regulates glucose and lipid metabolism and epithelial-mesenchymal transition (EMT) through control of TGF-β-Smad3 signaling. Kidneys from diabetic SIRT3 eKO mice were found to display significantly higher levels of FSP-1, aSMA, and TGFR1 in CD31-positive cells than those from diabetic littermate controls. SIRT3 deficiency in ECs leads to higher levels of TGF-β-smad3 signaling. It shows metabolism-related endothelial-to-mesenchymal transition (EndMT) defects, disrupting EC homeostasis and thus aggravating the process of fibrosis, which is one of the fibrotic phenotypes leading to PKC activation and PKM2 tetramer-to-dimer interconversion in diabetes (Sriyastava et al., 2021). SIRT6 is also implicated in the development of renal fibrosis. Cai et al., 2020 reported that SIRT6 deacetylates histone H3K56, reducing the expression of FN and MMP7 in β-catenin target genes. This suggests that the WNT/β-catenin signaling pathway may be a promising therapeutic avenue for treating renal fibrosis. Furthermore, SIRT6 was found to have some therapeutic effect on tubulointerstitial inflammation and renal fibrosis induced by unilateral ureteral obstruction (UUO) by regulating acetylation of β-catenin and extracellular matrix promoter enrichment at β-catenin acetylation sites (Jin et al., 2022). In addition, SIRT6 reduces inflammation by negatively regulating NF-κB signaling and synergistically regulates chronic renal fibrosis in ureteral obstruction (You et al., 2019).

A study has reported that renal (I/R) injury causes a decrease in p53 expression through the deacetylation of p53 by SIRT1, which subsequently promotes p53 ubiquitination and proteasomal degradation. Consequently, targeting the activation of SIRT1 and deacetylation of p53 may be a viable therapeutic approach to mitigate premature renal failure and delay the progression of CKD following AKI (Fan et al., 2013; Fu et al., 2022). Prior research has also shown that SIRT1 can alleviate sepsis-associated AKI (SA-AKI). HMGB1, a critical inflammatory mediator in sepsis pathogenesis, can be inhibited by SIRT1 through the deacetylation of its K28, K29, and K30 lysine sites, thereby hindering its transfer from the nucleus to the cytosol, suppressing the transmission of downstream inflammatory signals, and ameliorating renal function. These findings suggest a novel therapeutic strategy for the treatment of SA-AKI (Wei et al., 2019).

According to emerging evidence, mitochondrial function is thought to play a crucial role in kidney injury and repair following AKI (Lan et al., 2016; Zhang et al., 2021). Genetic deletion of one allele of SIRT1 significantly aggravated the tubular injury and apoptosis in an ischemia-reperfusion injury-induced AKI model (Fan et al., 2013). Further studies showed that SIRT1 activates PGC-1a, an important driver of renal protection in AKI (Tran et al., 2016), and activation of mitochondrial biogenesis and oxidative respiration by oxidative phosphorylation leads to proximal tubule repair (Funk and Schnellmann, 2013). Marina et al. found that improving mitochondrial dynamics by enhancing SIRT3 has the potential to be an approach to improving and preventing AKI. Cisplatin-induced severe mitochondrial damage in AKI mice was associated with decreased SIRT3 in the proximal tubules. In mechanistic studies, reduction of SIRT3 levels was found to initiate recruitment of the split protein Drp1 on the mitochondrial membrane, as well as downregulation of expression of the pro-fusion dynamin-related protein OPA1, ultimately facilitating mitochondrial fission and changing mitochondrial dynamics (Morigi et al., 2015). Further studies have found that mesenchymal stromal cells (MSCs) have renoprotective and regenerative driving forces after injury. Human umbilical cord (UC)-MSCs transplanted into cisplatin-induced AKI mice modulated the biogenesis of proximal tubule mitochondria by increasing PGC1α expression, NAD + biosynthesis, and SIRT3 activity, thereby promoting antioxidant defense and ATP production (Perico et al., 2017). In a rat model of renal I/R injury, the number of kidney mitochondria was significantly reduced due to accompanying structural changes (Szeto et al., 2017). GCN5L1, a newly identified acetyltransferase, was found to regulate mitochondrial protein acetylation and various mitochondrial biological functions. The expression of GCN5L1 was found to be significantly increased in AKI, and knockdown of GCN5L1 was found to reduce I/R-induced renal injury. GCN5L1 was found to acetylate TFAM at its K76 site, impairing its binding to TOMM70 and affecting its entry into mitochondria and DNA-binding ability, leading to mitochondrial dysfunction (Lv et al., 2022). GCN5L1 may serve as an energy sensor and modulator of mitochondrial function and, therefore, represents a potential target for intervention in DKD. In renal tissue from patients with DKD and mouse models, as well as in renal tubular epithelial cells (TECs) treated with high glucose, the expression of GCN5L1 was significantly increased. Treatment of STZ-DKD mice with sh-GN5L1AAV reduced tubulointerstitial injury and knockdown of GCN5L1 in TECs similarly reduced epithelial-to-mesenchymal transition (EMT) and inflammation, suggesting that targeting GCN5L1 may be an attractive therapeutic option for DKD. Moreover, the downregulation of GCN5L1 was found to protect the kidney through the MnSOD/ROS pathway *in vivo*. Downregulation of GCN5L1 was found to reduce acetylation levels of MnSOD K68, thereby attenuating oxidative stress-induced renal inflammation and fibrosis. GCN5L1 was found to aggravate oxidative stress-induced kidney injury by mediating MnSOD acetylation, indicating that GCN5L1 may be a potential intervention target for the treatment of DKD (Lv et al., 2021). In streptozotocin rats, stimulation with sodium butyrate (NaB) had a therapeutic effect on the kidney, with decreased HDAC activity, indicating that this protection was mediated by regulating histone acetylation, particularly in the context of DKD (Khan and Jena, 2014).

In numerous renal diseases, HDAC is expressed, with HDAC-3 playing a particularly crucial role in kidney development due to its unique structure. HDAC-3 hinders the proliferation of renal cells by inhibiting p27Kip1 (Sharma et al., 2009; Zhang and Cao, 2021). Recently, a novel anti-aging gene, *NM_026333*, was identified, and it appears to prevent renal aging by inhibiting the autophagy protein Atg7, which is related to HDAC-3 (Osanai et al., 2018). The kidney’s regenerative capacity after transient ischemia makes it an optimal organ for exploring I/R mechanisms. Ischemia induces histone remodeling and is involved in renal recovery following reperfusion. In proximal tubular cells, renal ischemia may lead to decreased HAT activity, which lowers histone acetylation levels. However, after reperfusion, the acetylation levels of histones are partly restored by downregulating HDAC-5. BMP7, a significant factor in kidney development, is involved in the regeneration process of renal tubules to repair kidney injury, and the reduced expression level of HDAC-5 causes the induction of BMP7 expression in proximal tubules (Gould et al., 2002; Villanueva et al., 2006; Marumo et al., 2008).

A recent genome-wide association study conducted by Chen et al. has revealed the involvement of DPF3 in the development of renal clear cell carcinoma (ccRCC). Upregulation of DPF3a expression is observed in ccRCC, and DPF3a specifically interacts with SNIP1 to form a complex with SMAD4, a key transcriptional regulator of the TGF-β signaling pathway, and p300 HAT. This complex activates p300 by binding SNIP1 and inhibiting its activity, which leads to increased acetylation of local histones. Ultimately, this activation of transcription results in cell migration, thus promoting ccRCC metastasis. These findings suggest that DPF3 may serve as a potential therapeutic target for ccRCC (Cui et al., 2022a).

## Glycosylation

**FIGURE 3:**
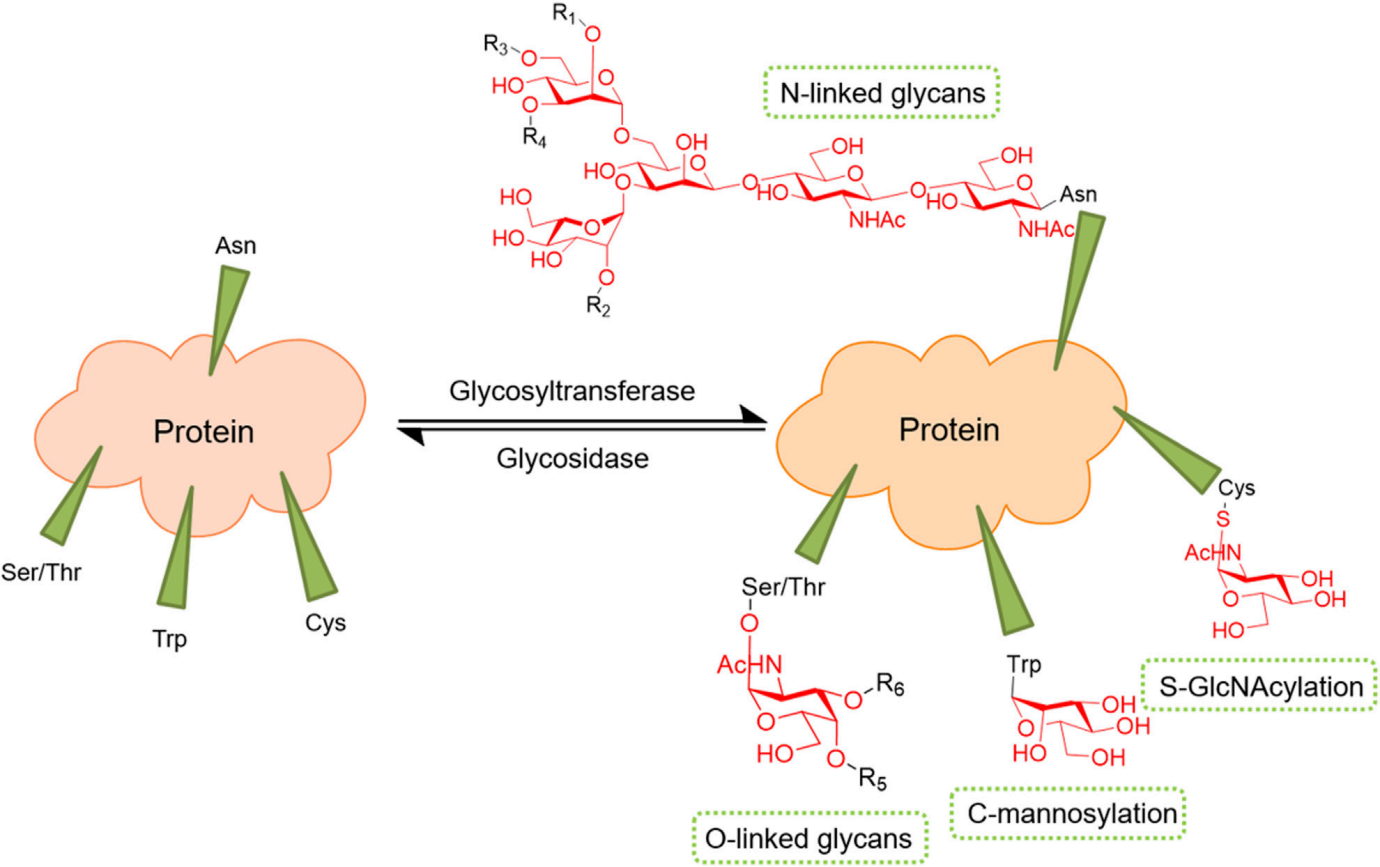
The process of protein glycosylation.

Protein glycosylation plays an important role in protein secretion, stability, binding, folding, and activity and is one of the most important PTMs of proteins. Protein glycolsylation includes the addition of N-linked glycans, O-linked glycans, phosphorylated glycans, glycosaminoglycans, and glycosylphosphatidylinositol (GPI) anchors to the peptide back as well as C-mannosylation of tryptophan glycosylation residues (Reily et al., 2019). Glycosylation of proteins is catalyzed by glycosyltransferases (GTs), which transfer sugars from donors to acceptors. In protein glycosylation, receptors are proteins or sugars already attached to proteins. Sugar donors are activated nucleotide sugars or phospholipid-linked sugars. According to the structural fold of GT pairs, they can be divided into one of three superfamilies: GT-A, GT-B, or GT-C (Figure 3). In fact, changes in glycosylation can modulate inflammatory responses, allow viral immune escape, promote cancer cell metastasis, or regulate apoptosis; the composition of glycome also impacts kidney function in health and diseases (Reily et al., 2019).

Abnormal glucose metabolism in DKD patients may lead to abnormal glycosylation, thereby driving DKD progression (Reily et al., 2019; Inagi, 2021). Extracellular ribonucleoside triphosphate diphosphate hydrolase 5 (ENTPD5) is an endoplasmic reticulum (ER)-located nucleotide hydrolase that hydrolyzes UDP to UMP, mediated by UGGT and promotes the correct folding of N-glycoproteins in the ER (Fang et al., 2010). Xu et al. found that ENTPD5 was mainly expressed in the renal tubules of the kidneys, and the expression level of ENTPD5 was changed in the late stage of DKD in diabetic mice and patients, first increasing and then decreasing. ENTPD5 has been reported to be an indicator for the clinical judgment of the pathological stage of DKD. More importantly, mechanistic studies in DKD have shown that hyperglycemia activates the hexosamine biosynthesis pathway (HBP), which promotes or inhibits SP1 O-glycosylation through a negative feedback mechanism, thereby regulating ENTPD5 expression at the transcriptional level. ENTPD5 regulates the N-glycosylation of unfolded proteins in the ER and promotes the proliferation or apoptosis of tubular epithelial cells. Interestingly, ENTPD5 has also been shown to be involved in the progression of other renal diseases by Xu et al., 2023 In a mouse model of UUO-induced nephropathy, multi-point injection of AAV-ENTPD5 and AAV-SH-ENTPD5 into the renal cortex upregulated and downregulated the expression of ENTPD5, and the results showed that UUO mice with overexpressed ENTPD5 had decreased creatinine and blood urea nitrogen levels, significantly improved renal morphology, reduced renal interstitial fibrosis, and decreased tubular cell apoptosis. In addition, Ankita et al. discovered the role of long non-coding RNAs (lncRNAs) in DKD with glycosylation, providing new ideas for exploring the remission of glycosylation-related diabetic nephropathy (Durge et al., 2022). IgAN is the most common primary glomerulonephritis worldwide and is characterized by the deposition of polymerized Gd-IgA1 on the mesangium in the form of immune complexes. Most IgAN patients have elevated Gd-IgA1 serum levels (Moldoveanu et al., 2007; Berthoux et al., 2012). Aberrant glycosylation of IgA1 O-glycans is present in genetically susceptible individuals and autoantigenic epitopes that can be recognized by IgG autoantibodies (Huang et al., 2016; Suzuki and Novak, 2021), leading to the formation of ICs containing Gd-IgA1 and renal deposition, which leads to glomerular injury (Lai et al., 2016; Suzuki and Novak, 2021). Nephrin is reported to be a membrane glycoprotein expressed on the surfaces of podocytes, where it functions as an adhesion and scaffold receptor and signaling molecule. N-glycosylation at up to 10 potential glycosylation sites is crucial for the proper folding, transport, surface expression, and function of nephrin. Indeed, N-glucan deficiency in neparin leads to poor septal cleft formation and impaired renal function (Reily et al., 2019). This evidence suggests that glycosylation impacts aberrant cell signaling, transcription factor activation, and alterations in gene expression patterns, in addition to affecting protein structure and function.

In addition, the final products formed by glycosylation are called glycation end products (AGEs). AGEs have been reported to play an important role in the etiology of kidney injury in diabetic nephropathy. The severity of diabetic nephropathy is closely related to the number of AGEs and the expression of advanced glycation end product receptors (RAGE) in the glomerular and tubulointerstitial compartments. AGEs are thought to be a factor in decreased renal filtration rate (GFR) in diabetic nephropathy. AGEs promote the synthesis of asymmetric dimethylarginine (ADMA), which is inversely correlated with endothelial function. In addition, AGEs decreased mRNA levels of the ADMA-degrading enzyme dimethylarginine dimethylaminohydrolase (DDAH-II) and increased ADMA levels, and the antioxidant N-acetylcysteine inhibited the development of these phenomena. The results suggest that AGE-RAGE-mediated ROS production may be associated with endothelial dysfunction in diabetic end-stage renal disease patients and may promote ADMA formation by decreasing DDAH activity in endothelial cells (Ando et al., 2013; Khanam et al., 2023). In addition, AGEs affect extracellular matrix (ECM)metabolism and contribute to the development of diabetic nephropathy. Diabetic nephropathy is characterized by ECM accumulation in the glomerular mesangium, tubulointerstitium, and glomerular basement membrane. AGEs affect collagen metabolism and interfere with extracellular matrix and cell-matrix interactions, leading to the loss of the epithelial phenotype. Accumulation of AGEs in extracellular matrix components aggravates glomerulosclerosis, and glycosylated collagen in basement membranes promotes platelet aggregation (Marshall, 2016). AGEs promote expression of TGF-β and promote fibrosis in podocytes, tubular epithelial cells, and mesangial cells (Coughlan et al., 2007; Khanam et al., 2023). Briefly, AGEs caused changes in ECM and the appearance of a late renal phenotype. Interestingly, AGEs have been found to bind to their specific RAGE and can activate NF-κB signaling to promote an increase in the level of the inflammatory factor TNF-α. Meanwhile, the AGE-RAGE axis promotes the production of ROS by activating NADPH oxidase and, through other similar mechanisms, leads to aggravated oxidative damage to cells and promotes fibrotic responses in diabetic nephropathy (Tobon-Velasco et al., 2014; Cannizzaro et al., 2017). More interestingly, Li et al. explored the effect of flavonoids from buckwheat husk extract on AGEs. They synthesized two different types of AGEs, including the BSA-MGO model and the BSA-Glu model, to explore the effects of total buckwheat husk flavonoids (TBHF) as well as each monomer compound on these two models. The results demonstrated that both AGEs could be effectively mitigated in all experimental groups, with a stronger effect observed on the BSA-MGO model. The flavonoid monomer component had a more significant and effective breaking effect than TBHFs. At the same time, mouse experiments have confirmed a more significant inhibitory effect on the AGE-RAGE signaling pathway (Li et al., 2021).

## Phosphorylation

**FIGURE 4:**
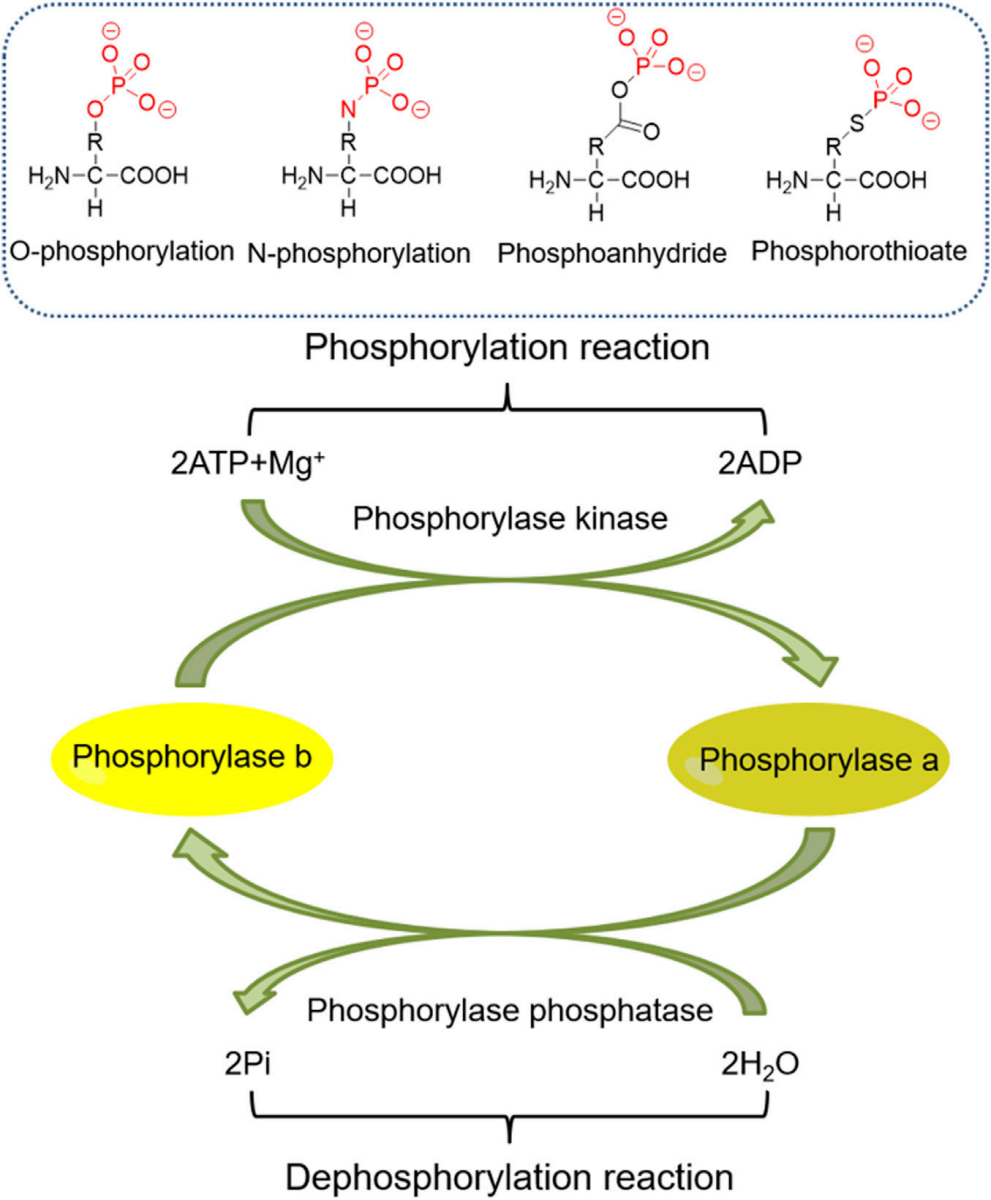
The process of protein phosphorylation.

Phosphorylation has long been an important PTM of proteins, and its role in kidney diseases has also gained increasing attention. Here, we briefly present studies on the role and molecular mechanisms of histone phosphorylation and non-histone phosphorylation in kidney diseases (Figure 4).

Sixteen histone residues have been reported to be phosphorylated in mammalians (Banerjee and Chakravarti, 2011). Histone phosphorylation is involved in many aspects of chromatin function, which include transcriptional activation and repression, chromatin condensation, and DNA repair. Phosphorylation of serine 10 and 28 of H3 and serine 32 of H2B is associated with regulating epidermal growth factor (EGF)-responsive gene transcription, while EGF and its receptor play an important role in renal regeneration after AKI. It has been found that inhibition of H3 phosphorylation by PD98059, a selective mitogen-activated protein kinase 1 inhibitor, or poly (ADP-ribose) polymerase inhibition with 3-aminobenzamide, improves tubular cell survival (Tikoo et al., 2001). Phosphorylation of histone H2AX at Ser139 by ATM in mammalian cells is a biochemical hallmark of the DNA damage response (DDR). Phosphorylated H2AX, also known as γH2AX, significantly increased γH2AX-positive tubular epithelial cells in ischemia-reperfusion cortical tissues (Ma et al., 2014). Histone phosphorylation modifications are also important in DKD. DKD is an inflammatory disease and an endothelial disease. The receptor CCR2 expressed by GECs was found to bind to ligands and induce upregulation of expression of the proinflammatory adhesion molecule VCAM-1 through a pathway dependent on the regulation of H3Ser10 phosphorylation of MSK1/2. Histone H3Ser10 levels are elevated in experimental and human DKDs, which allows us to continue to explore therapeutic targets for DKD from a new perspective (Alghamdi et al., 2018).

Lin et al. explored the mechanism of FADD phosphorylation in renal fibrosis in FADD-D mice (Hua et al., 2003). Phosphorylation of FADD has been found to activate the TLR4/myD88/NF-κB, mTOR, and TGF-β/Smad signaling pathways. First, activation of the TLR4/myD88/NF-κB pathway was detected by quantitative polymerase chain reaction and western blotting in FADD mice. The expression levels of inflammatory cytokines TNF-α, IL-6, and TNF-β were significantly increased, and the degree of macrophage infiltration was significantly increased compared with those in controls. In addition, protein levels of P70S6K, as well as phosphorylation levels of mTOR, GSK3β, and AKT, were significantly increased in FADD-D mice. Immunofluorescence staining showed that expression levels of myofibroblast markers such as Snail, N-cadherin, vimentin, and fibronectin were significantly increased compared with those of control mice, and these data suggest that FADD phosphorylation promotes EMT as well as pro-fibrotic factor expression through the mTOR pathway. Interestingly, knockdown or overexpression of FADD in MES cells and HK2 cells showed increased expression levels of α-SMA and TGF-β1 and decreased expression of E-cadherin, activating the TGF-β1 pathway and promoting the process of EMT. This evidence led us to understand that phosphorylation of FADD may lead to IgA nephritis and, ultimately, renal fibrosis (Lin et al., 2023). In addition, Li et al. first demonstrated increased phosphorylation of microtubule-associated protein 4 (MAP4) in urine samples from diabetes patients and in streptozotocin (STZ)-induced diabetic mouse kidneys. MAP4 phosphorylation induces tubulin (MT) and F-actin rearrangement, podocyte EMT, and apoptosis, leading to proteinuria, similar to the processes in diabetic nephropathy. Blocking p38/MAPK signaling inhibited podocyte differentiation and apoptosis. These results suggest that modulation of p38/MAPKMAP4 phosphorylation signaling may identify a novel therapeutic target to attenuate proteinuria and renal fibrosis in patients with diabetic nephropathy (Li et al., 2022). ER stress has been reported to be associated with podocyte apoptosis in diabetic nephropathy. Zhang et al., 2017 found that cyclin-dependent kinase 5 (Cdk5) plays an important role in its mechanism. High-glucose stimulation was found to rapidly induce upregulation of expression of GPR78, an ER stress marker, while tunicamycin (TM), an ER stress inducer, promoted Cdk5 expression in podocytes. Importantly, CDK5 phosphorylates MEKK1 at Ser280 in TM-treated podocytes, and they increase JNK phosphorylation. In addition, blocking this pathway could reduce TM-induced podocyte apoptosis. These results suggest that Cdk5 plays an important role in ER stress-induced podocyte apoptosis in diabetic nephropathy through the MEKK1/JNK pathway. In addition, Cdk5, as a key kinase, can participate in mitochondrial dysfunction and podocyte injury by promoting Sirt1 phosphorylation at S47, which leads to the progression of diabetic nephropathy (Wang et al., 2021). Interestingly, sulfides have been found to have some protective effects in the kidney in recent years, and Sun et al. explored the mechanism of action of NaS in renal injury and found that Na2S4 ameliorates diabetes-induced renal injury by sulfidation and upregulation of SIRT1 protein expression, followed by inhibition of p65 NF-κB/STAT3 phosphorylation and acetylation (Sun et al., 2021).

This evidence leads us to understand the role and molecular mechanism of protein phosphorylation in kidney diseases, and PTMs have gradually become a new idea for treating kidney diseases, providing more options for treatment.

## Crotonylation

Histone lysine crotonylation (Kcr), an evolutionarily-conserved histone mark, involves a modification of the addition of crotonyl moieties from crotonyl-CoA to lysine residues, leading to changes in the charge of histones (Tweedie-Cullen et al., 2012; Fontecha-Barriuso et al., 2018). Kcr, a novel PTM, was initially identified in human cell lines and mouse sperm histones (Peng et al., 2011). It is present in all core histones and acts as a marker for active promoters and potential enhancers (Tan et al., 2011). In a previous study, the role of acetylation modification in various renal diseases and renal cancer was reviewed, highlighting its significance in kidney function. Although histone crotonylation and histone acetylation share enzyme modulators and structurally similar modifier groups, they differ in function and mechanism. Crotonylation is essential for mammalian cell transcription and is not redundant with acetylation (Tan et al., 2011; Wei et al., 2017). The transferases involved in crotonylation include HCT, P300/CBP, PCAF, and MOF, while those involved in descrotonylation are HDAC-1, HDAC-2, HDAC-3, HDAC-8, SIRT1, SIRT2, and SIRT3 (Wan et al., 2019). In this review, we present the current understanding of the role of lysine crotonylation in renal diseases and the associated molecular mechanisms.

AKI and CKD represent severe and closely interconnected consequences of kidney injury, given that CKD can increase the likelihood of AKI and, in turn, worsen CKD progression. Renal injury is currently considered the most prominent disease for assessing the function and extent of histone crotonylation, which has been observed to increase during AKI (Justo et al., 2006). Notably, the therapeutic potential of crotonate in treating AKI-induced kidney injury-has been attributed to its ability to enhance histone crotonylation. CCL2, a chemokine known to promote renal inflammation, can be countered by the protective effects of PGC1a and SIRT3 on the kidney (Ruiz-Andres et al., 2016; Fontecha-Barriuso et al., 2019; Martinez-Moreno et al., 2020a). *In vitro* cell experiments conducted on mouse tubular epithelial cells have shown that crotonate treatment induces an increase in histone crotonylation, thereby promoting increased expression of PGC1a and SIRT3 and decreased expression of CCL2 (Martinez-Moreno et al., 2020b). These findings suggest that histone crotonylation plays a favorable role in mitigating renal injury; however, the therapeutic efficacy of histone crotonylation in treating DKD requires further investigation.

Huang et al., 2021a have identified that class B scavenger receptor CD36 is strongly associated with renal failure based on the KEGG pathway enrichment analysis. CD36 is expressed in various renal cells, including the proximal tubular epithelial cells, mesangial cells, podocytes, monocytes, and macrophages. It plays a pivotal role in diverse biological processes such as lipid accumulation, inflammation, energy reprogramming, apoptosis, and renal fibrosis (Yang et al., 2017). Notably, histone crotonylation has been shown to influence the expression of CD36-related genes, and modulating histone and non-histone crotonylation may have therapeutic potential for treating patients with chronic renal failure (CRF) by slowing disease progression and restoring these functions.

## 2-hydroxyisobutyrylation

2-hydroxyisobutyrylation (Khib), a PTM that utilizes 2-hydroxyisobutyrate (HIBA) and 2-hydroxyisobutyryl-CoA (HibCoA) as substrates, is conserved across eukaryotic and prokaryotic cells and is involved in various biological processes (Dai et al., 2014; Huang et al., 2018a). The catalytic enzymes for this modification are P300 and Tip60, while HDAC1-3 and SIRT3 are responsible for its removal (Huang et al., 2018b; Wang et al., 2022b). During kidney development, epigenetic regulation plays a crucial role, with Khib, in addition to DNA methylation and H3K27me, also contributing to this process. SIRT3, functioning as a de-2-hydroxyisobutyrylase, is highly expressed in early kidney development, with increased histone lysine H3 and H4 Khib promoting glycolytic processes in SIRT3-null mice. However, after AKI, Khib may lead to renal function impairment or even death (Perico et al., 2021). Khib has also been found to play a role in important cellular processes such as glycolysis/gluconeogenesis and the tricarboxylic acid cycle (TCA) cycle. A recent study by Huang et al., 2021b in 2021 has shown that Khib mainly accumulates in the interleukin (IL)-17 signaling pathway and phagosome category, which is associated with IgAN. As renal fibrosis is a common marker of various etiologies, including CKD and ESRD, understanding its pathogenesis is particularly important. The Rho/Rho-Kinase (Rho/ROCK) signaling pathway, which is significantly upregulated in ESRD, is found to be modified by Khib, and through this pathway, this modification may promote renal fibrosis in mesangial cells (Zheng et al., 2021). Although this sheds light on the role of Khib in renal diseases, more research is necessary to elucidate the impact of histone modifications in the context of other renal diseases.

## β-hydroxybutyrylation

β-hydroxybutyrate (BHB) is the predominant ketone body, contributing to 70%–80% of the overall ketone pool. It is biosynthesized by the hepatic metabolism of fatty acids under conditions of diminished glucose levels and energy demand in the body (Anson et al., 2003). In the cellular milieu, increased levels of BHB promote lysine β-hydroxybutyrylation (Kbhb) on histones, thereby demonstrating an association of histone Kbhb marking with active gene promoters of ketoacid-induced metabolic pathways. The discovery of histone Kbhb as an innovative epigenetic regulator of cellular physiology and pathology has garnered significant attention (Xie et al., 2016). The enzymatic catalysis of BHB at lysine residues is facilitated by CBP and p300, whereas SIRT13 and HDAC1-3 are involved in its removal. However, further investigation is warranted to identify the histone Kbhb-specific “eraser” (Zhao et al., 2018; Chen et al., 2020; Zhou et al., 2022a).

Although ketosis is known to be detrimental to individuals with diabetes, BHB at physiological concentrations below 10 mM may play a crucial protective role. Moderately elevated ketone levels have been closely linked to increased insulin sensitivity in diabetes. Studies have demonstrated that feeding mouse models with type 1 (Akita) and type 2 (db/db) diabetes with a ketogenic diet for 8 weeks reduces blood glucose levels, urine protein/creatinine ratio, and oxidative stress-related gene expression (Poplawski et al., 2011). The use of BHB has also been found to reduce diabetic retinopathy (Min et al., 2018; Luo et al., 2020a). Although the relationship between BHB and DKD has been explored to a lesser extent, the potential beneficial effects of Kbhb in DKD have been suggested. Elevated serum BHB levels have been found in both fasted and streptozotocin-diabetic mice, and this increase in BHB is associated with an increase in histone Kbhb levels, possibly due to histone Kbhb’s role in energy metabolism reprogramming (Xie et al., 2016). Studies have further revealed that BHB may have a protective effect against oxidative stress in the mouse kidney by inhibiting the activities of HDAC-1 and HDAC-2 and inducing histone acetylation at the promoter genes (*Foxo3a* and *Mt2*) of anti-oxidative stress (Shimazu et al., 2013). The expression of anti-oxidative stress genes, such as *Duox1* and *SOD1*, may be increased in renal disease, thereby improving -DKD-in type 1 (Akita) and type 2 (db/db) diabetic mice, and BHB may play a role in this regard. Although it has not been verified in renal cells, BHB has been found to protect neuronal cells from glucose-induced oxidative stress (Obokata et al., 2017). Studies have also demonstrated the therapeutic effect of BHB on glomerulosclerosis in diabetic rats, as it ameliorated glomerulosclerosis by increasing the expression level of matrix metalloproteinase-2 (MMP-2) and causing an increase in H3K9bhb at the MMP2 promoter in the kidney of diabetic rats, thus alleviating the morphological changes of glomeruli and glomerular type IV collagen content (Luo et al., 2020a).

Prior research has revealed that dietary restriction is linked to reduced renal cyst growth and mTOR activity in mouse models of PKD (Kipp et al., 2016). Recent investigations have established that time-limited feeding (TRF) can lower mTORC1 and STAT3 signaling, as well as alleviate interstitial fibrosis and proliferation in cystic kidneys. Additionally, oral administration of BHB has been found to delay the advancement of PKD in juvenile rats, suggesting that BHB could potentially influence the progression of PKD through specific molecular mechanisms (Torres et al., 2019; Luo et al., 2020a).

## Propionylation and butyrylation

In 2007, Chen et al. reported the discovery of two novel *in vivo* lysine modifications in histones: propionylation (Kpr) and butyrylation (Kbu). Through *in vitro* labeling and mass spectrometry peptide mapping, it was confirmed that acetyltransferase p300 and CREB binding proteins catalyze the lysine propionylation and lysine Kbu of histones. The acyl groups responsible for Kpr and Kbu originate from propionate and butyrate, respectively, and are converted into small active molecules similar to acetyl-CoA via ACSS2. These molecules participate in the mediation of histone acylation processes (Chen et al., 2007).

In 2019, Fabian and colleagues reported the potential benefits of propionate supplementation in reducing systemic inflammatory response and protecting against ESRD in patients (Meyer et al., 2020a). Contrast-induced nephropathy (CIN) can lead to AKI and inhibit the activation of NF-κB even after NaB treatment, resulting in inflammatory response and tubular injury (Machado et al., 2012). In a rat model of AKI induced by I/R, pre-conditioning with butyric acid significantly improved renal function and reduced serum creatinine levels, thus mitigating I/R-induced renal damage (Sun et al., 2022). Zhou et al. discovered that intraperitoneal injection of NaB could alleviate dyslipidemia in DKD mice, maintain glucose and lipid homeostasis through non-gastrointestinal intervention, and improve renal injury caused by DKD. Furthermore, their study revealed that histone H3K9bu was significantly up-regulated by NaB, which played a role in reversing anti-inflammatory and anti-fibrotic effects and improving renal injury caused by DKD (Zhou et al., 2022b).

## Succinylation

In 2010, Zhang et al. reported the first identification and validation of lysine succinylation (Ksucc) in *Escherichia coli* proteins. This modification entails the transfer of a succinyl group to a lysine residue in the protein and is a reversible and dynamic process that is evolutionarily conserved (Zhang et al., 2011). Subsequent investigations have comprehensively explored succinylation in bacterial and mammalian cells, revealing its widespread occurrence in various mitochondrial metabolic enzymes (Park et al., 2013) and its association with diverse diseases, including the liver, heart, and lung diseases (Sadhukhan et al., 2016). Notably, Ksucc has also been implicated in the pathogenesis of renal disease. Studies have revealed a significant increase in succinate levels in the cytosol during I/R in the I/R rat model, closely linked with reactive oxygen species generation (Kamarauskaite et al., 2020). Furthermore, the accumulation of succinate in the diabetic kidney has been shown to inhibit mitochondrial fatty acid oxidation dysregulation and promote the formation of ROS (Wang et al., 2022c). SIRT5, a crucial eraser, regulates protein succinylation. In ccRCC, SIRT5 can synchronously inhibit succinylation of the succinate dehydrogenase (SDH) complex subunit A (SDHA) to promote the proliferation of ccRCC cells, thus providing a novel avenue for the treatment of ccRCC (Meyer et al., 2020a).

## Malonylation

Chao et al. have successfully validated the conserved PTM, malonylation (Kma) in mammalian and bacterial cells. They identified SIRT5 as a key regulatory enzyme for both lysine malonylation and lysine succinylation and demonstrated its ability to catalyze lysine desmalonylation and desuccinylation reactions both *in vitro* and *in vivo* (Peng et al., 2011). Through functional enrichment analysis, they have revealed significant enrichment of Kma in glucose and fatty acid metabolic pathways (Du et al., 2015). Moreover, their investigations indicate that histone malonylation levels are elevated in NE4C of high glucose-treated mice, suggesting the potential involvement of histone malonylation in neurological complications of diabetes. The authors have also established a close relationship between protein malonylation and energy metabolism processes, such as glycolysis and mitochondrial respiration. SIRT5 deficiency in chondrocytes results in elevated malonylation and alters cellular metabolic processes, particularly related to the TCA, glycolysis, and amino acid cycles (Zhu et al., 2021a). Interestingly, Judy et al. found reduced malonylation levels in the renal cortexof type 2 diabetic BKS db/db mice, which correlated well with increased SIRT5 expression. Proteomic analysis showed that the malonylation levels of aldolase A and aldolase B, targets of the glycolytic pathway, were significantly decreased in the db/db cortex, as well as the malonylation levels of PGM1, PFKM, LDHA, LDHB, and other enzymes in the glycolytic pathway. At the same time, the malonylation levels of SLC27A2, CAT1, ACOX1, DBP, LBP, SCP2, and other enzymes in the peroxisomal fatty acid oxidation pathway were also decreased in diabetic db/db cortices. Interestingly, increases in SIRT5 levels and decreases in malonylation were found to be associated with increases in peroxisomal FAO. Importantly, investigators also analyzed diabetic kidney transcriptome data from a West South American Indian cohort and showed tubulointerstitial-specific increases in SIRT5 expression. These data allow us to gain further insights into the potential role of SIRT5 in the metabolic reprogramming of DKD, and its role in propionylation makes SIRT5 a potential target for the treatment of DKD (Baek et al., 2023). Additionally, the authors investigated the role of malonic acid acylation in renal I/R injury and found that inhibition of the mitochondrial enzyme SDH using malonate prodrugs can effectively improve renal injury caused by ROS production during ischemia. In summary, these findings suggest that malonylation plays a critical role in various cellular processes and disease states, particularly DKD, and warrants further investigation (Beach et al., 2020).

## Lactylation

In 2019, Professor Yingming Zhao and his research team at the University of Chicago identified a novel form of post-translational protein modification known as lysine lactylation (Kla), which involves adding a lactate group to lysine residues of nuclear histones. Histone lactylation has been shown to play a role in regulating tumorigenesis by activating gene transcription and inflammation through macrophages (Jiang et al., 2021; Xie et al., 2022). Further studies have since demonstrated that lactylation is a crucial mechanism for lactate to exert its biological functions, including regulation of glycolysis-related cellular functions (Li et al., 2020), macrophage polarization (Irizarry-Caro et al., 2020), nervous system regulation (Hagihara et al., 2021), and development in rice grains (Meng et al., 2021). Lactylation has been implicated in numerous physiological effects and linked to the pathophysiology of several diseases, including renal disorders. Specifically, lysine lactylation is induced by lactic acid, and the H3K18la modification has been found to be highly enriched at the promoter of the platelet-derived growth factor receptor β (PDGFRβ). Lactylation modification activates the transcription of PDGFRβ, thereby promoting tumor proliferation and migration in ccRCC (Yang et al., 2022).

In 2021, Cui et al., 2021 revealed that lactate could stimulate the lactylation of histones via p300 in lung myofibroblasts, which consequently facilitates the profibrotic response of macrophages, leading to the advancement of pulmonary fibrosis. Recent studies have demonstrated that lactate is not merely a “waste product” of glycolysis, as it is capable of regulating both innate and adaptive immune cells and causing considerable changes in gene expression (Martinez-Outschoorn et al., 2011; Haas et al., 2016). Despite this, renal lactylation has not been fully explored. During the onset of AKI, the clearance of lactic acid in the kidney declines, resulting in its accumulation in the bloodstream. Subsequently, this accumulated lactic acid contributes to the recovery process of AKI (Wen et al., 2021). The precise mechanisms and functions of lactylation in renal diseases necessitate further investigation. Renal diseases are commonly associated with significant pathogenic factors such as ischemia and hypoxia. In the context of DKD, there is evidence of elevated lactate production, which merits further exploration in terms of the potential impact of lactylation on DKD. Further research is therefore warranted to elucidate the role of lactylation in DKD (Lin et al., 2011).

Previously, relevant studies on the role and molecular mechanisms of acetylation in renal diseases were reviewed. Subsequent investigations have shown that P300, traditionally known as a HAT, is also overexpressed and capable of decreasing the expression in HEK293T and HCT116 cells (Zhang et al., 2019), thereby highlighting its potential as a “writer” of lactylation. However, the potential association between lactylation and acetylation remains unclear. Notably, histone lactylation levels increased at 16–24 h during M1 macrophage polarization, while acetylation levels decreased at the corresponding time nodes after lipopolysaccharide and interferon-gamma treatment of bone marrow-derived macrophages (Zhang et al., 2019). Thus, a possible divergence between lactylation and acetylation may exist. Consequently, given the possibility of lactylation and acetylation playing a pivotal role in the advancement of renal diseases, further investigations are required.

## Histone methylation

Histone methylation is a vital process that involves the transfer of methyl groups to histone lysine or arginine residues by histone methyltransferases (HMTs). This process is critical for the regulation of gene expression and maintenance of genome stability, which require coordinated enzymatic regulations (Audia and Campbell, 2016). Histone methylation relies on HMTs, which can be classified into lysine-specific (KMTs) and arginine-specific (PRMT) methyltransferases (Black and Whetstine, 2013). The extent of methylation varies for different lysine and arginine residues, with lysine residues accepting up to three methyl groups to produce mono-, di-, or trimethyl-lysine, while arginine residues can accept up to two methyl groups to yield mono- or dimethyl-arginine. Reversible covalent modifications of histones are a crucial aspect of this process, and lysine-specific demethylase 1A (KDM1A, also known as LSD1) has been identified as the first demethylase to catalyze H3K4 and H3K9 demethylation. However, research on arginine demethylase is still in progress (Shi et al., 2004).

In recent years, there has been increasing evidence to suggest that histone modifications play a critical role in various pathological processes associated with diabetes, including metabolic memory, inflammatory response, and endothelial dysfunction. Among these modifications, histone methylation has emerged as a key player in the pathogenesis of DKD, along with acetylation. Several previous studies have established the involvement of various histone methylation modifications in the development of DKD, such as H3K4me1/2/3, H3K36me2/3, H3K79me2, H3K9me2/3, H3K27me3, and H4K20me3 (Zhong and Kowluru, 2010; Liao et al., 2018; Qu et al., 2020). Notably, H3K27 and H3K4 are two widely investigated histone methylation modifications (Sun et al., 2014). The former is targeted by two specific histone demethylases, UTX (also known as KDM6A) and JMJD3 (also known as KDM6B), with UTX being upregulated in podocytes of patients with DKD and focal segmental glomerulosclerosis. Studies have demonstrated that UTX modulates the pathogenesis of DKD in multiple ways, including promoting inflammatory responses and DNA damage, whereas overexpression of UTX in podocytes can upregulate Jagge-1 (Majumder et al., 2018). Furthermore, UTX overexpression in renal tubular and mesangial cells has been shown to improve early DKD lesions in animal models, indicating its potential as a therapeutic target for DKD (Chen et al., 2019). H3K4me3 is also implicated in DKD development, with PTIP, a component of the MLL3/4 histone-H3K4 methyltransferase complex, playing a critical role. Notably, the downregulation of DACH1 in podocytes has been shown to reduce DACH1-PTIP promoter binding, resulting in elevated H3K4me3 levels and increased susceptibility to podocyte injury (Cao et al., 2021).

Epigenetic regulation is a process that induces changes in gene expression without modifying the heritable changes in DNA, and it has a significant role in the pathophysiology of kidney transplantation. During kidney transplantation, I/R is a process that cannot be avoided (Debout et al., 2015). In cases of acute tubular necrosis in acute and chronic allograft injuries, the demethylation of the C3 complement gene, and the methylation of the CALCA (calcitonin-related polypeptide α) gene in the urine of renal transplant patients demonstrate that DNA methylation has potential therapeutic benefits in cold ischemia-related kidney transplantation injury (Mehta et al., 2006; Parker et al., 2008). The methylation of different T cells can alter immunoreactivity outcomes after kidney transplantation. In 2011, Bestard et al. discovered that demethylation of Foxp3 was associated with a high expression of Treg cells and rejection outcomes in kidney transplant patients. In 2020, it was demonstrated in mouse kidney transplantation that demethylation with DNA methyltransferase (DNMT) inhibitors and negative regulators that enhance the mTOR signaling pathway can decrease inflammatory damage and acute rejection in kidney transplantation (Zhu et al., 2021b).

Prior investigations have demonstrated that disruption and activation of endoplasmic reticulum proteostasis play a crucial role in the development of various kidney-related ailments, such as glomerulosclerosis, glomerulonephritis, ischemia, DKD, nephrotoxicity, and CKD (Liu et al., 2016; Cybulsky, 2017; Gu et al., 2018). Consequently, targeting the deficient cellular unfolded protein response could be a potential therapeutic strategy for halting or mitigating the progression of renal diseases. Recent studies indicate that histone H3K9 and H3K27 methylation can enhance ER stress, thus ameliorating oxidative stress and the concomitant pathological process of exacerbating kidney damage (Diaz-Bulnes et al., 2022).

## Potential therapeutic applications in kidney disease

PTMs are important candidate targets for the prevention and treatment of kidney diseases. In this review, we reveal the important role of PTMs in various renal diseases. We found that PTMs have multiple protective effects on the kidneys, including relieving oxidative stress and inflammatory responses, decreasing levels of apoptosis, increasing autophagy, promoting gene expression, and regulating ATP and mitochondrial homeostasis, indicating that PTMs are significant therapeutic targets for preventing kidney diseases. In this context, it is important to highlight the emergence of novel protein PTMs, such as lactation, which provide new ideas for treating kidney diseases. In addition, by summarizing the therapeutic targets of PTMs in renal diseases (Table 2), we found that most studies of PTMs are clinical studies, and therefore, the focus of PTM research needs to shift to clinical applications, including pharmacokinetic, pharmacodynamic, and safety studies of various transferases and detransferase agonists or inhibitors.

Epigenetic modifiers can serve as potential therapeutic targets for renal disease, and promising results have been obtained in randomized clinical trials. Many epigenetic modifying agents, such as histone modifiers and DNA methylation inhibitors, are currently available and have been tested in preclinical models of AKI and CKD. Increased histone acetylation using HDACis generally protects the kidney from AKI and promotes kidney repair. In addition, inhibition of DNA methylation by DNA methylation inhibitors or DNA demethylation activators also improves renal fibrosis (Fontecha-Barriuso et al., 2018). However, preclinical studies have shown that some epigenetic drugs, such as HDACis, have renoprotective effects at low doses, but nephrotoxicity at high doses, so there are still great limitations (Dong et al., 2008). Epigenetic markers, such as bromodomain and extra terminal (BET) proteins, also play a very important role in the treatment of renal diseases.The BD2 selective BET inhibitor apabetone was the first epigenetic modulator to conduct a phase 3 clinical trial in DKD with renal function as the end-point (Martinez-Moreno et al., 2020a). The apabetone trial in patients with T2DM and CKD responded well to treatment, with fewer hospitalizations and significantly fewer major adverse cardiovascular events (MACEs) (Nicholls et al., 2021). Losartan, a representative AT1R blocker used to treat clinical DKD, was found to partially reduce histone methylation observed in db/db mice, but the specific mechanism remains elusive (Reddy et al., 2014). In addition, substrate availability regulates histone posttranslational modifications, such as the aforementioned ability of ESRD patients to reduce systemic inflammatory responses after propionate supplementation and has some protective effect against ESRD (Meyer et al., 2020a). In 2021, Svetlana et al. reported that determination of urinary free amino acids and their PTM metabolites and AGEs in kidney transplant recipients (KTR) is a non-invasive method in kidney transplantation (Baskal et al., 2021). However, statistical significance in observational studies in nature is not equivalent to biological significance. It remains unclear whether the relationship between age and the excretion rate of PTM metabolites and mortality is causal or associative, and the selection of the study population also has some limitations. In addition, it is interesting to note the idea that acetylsalicylic acid (ASA) does not increase fibrinogen acetylation in T2DM patients and that glycosylation may block previously identified acetylation sites *in vitro* (Bryk et al., 2021). In addition, another limitation of epigenetic drugs is that they are highly non-specific and induce global epigenetic changes that are not gene-specific or organ-specific. Xu et al., 2018 found that novel high-fidelity methods for crispr-cas9 could achieve hydroxymethylation of specific genes. The fusion of the catalytic domain of the DNA methylation eraser TET3 with inactivated high-fidelity Cas9 (dHFCas9) creates a construct that specifically targets gene demethylation by guiding RNAs. The use of this approach resulted in successful reactivation of Rasal1 and Kl, leading to attenuation of UUO-related renal fibrosis. These studies have led us to realize that relevant clinical studies of PTMs in kidney disease still deserve our exploration.

Interestingly, we found that environmental factors also influence the role of PTMs in kidney disease. Fasting has been found to increase levels of 3-hydroxybutyrate, thereby increasing histone β-hydroxybutyrate, such as PPARGC1A gene encoding PGC-1α, which is a key renoprotective molecule (Fontecha-Barriuso et al., 2020; Zhang et al., 2020). A high-fat diet, sedentary lifestyle, or exposure to toxic substances, can lead to chronic metabolic inflammation causing hyperglycemia, thereby increasing the likelihood of DKD development (Naidoo et al., 2018; Ramos-Lopez et al., 2021). Epigenetic mechanisms control the expression of these inflammatory factors, such as crotonate increases histone crotonylation, PGC1a and SIRT3 expression, and CCL2 expression in tubular epithelial cells, which acts as a chemokine that promotes renal inflammation, and its decreased expression can alleviate renal injury at the onset of AKI (Naidoo et al., 2018; Martinez-Moreno et al., 2020b; Ramos-Lopez et al., 2021). In addition, TGF-β signaling stimulated histone acetylation and methylation through activation of HAT p300/CBP upon stimulation with high glucose, resulting in enrichment of H3K9/14Ac and HAT p300/CBP at renal fibrosis gene promoters. This further leads to increased fibrosis gene transcription and epithelial to mesenchymal transition (EMT) in DKD kidneys (Sun et al., 2017). This allows us to understand that genetic and external factors are involved in many kidney diseases and, in some cases, environmental changes lead to adaptive epigenetic changes. In general, epigenetic modifications are considered stable and heritable during cell division, and they may be reversible, in addition to environmental factors that may also be influenced by disease states and genomes. Up-regulation of p300/CBP-associated factor (PCAF; HAT) was found to be associated with increased acetylation of histones (e.g., H3K18Ac) and increased expression of inflammatory genes in a lipopolysaccharide (LPS) -induced septic AKI mouse model (Huang et al., 2015); down-regulation of the renoprotective factor Klotho (in mice encoded by Kl) in folate-induced AKI was shown to occur through TWEAK (TNF-atedrelinducer of apoptosis) -mediated deacetylation of the Kl gene promoter (Moreno et al., 2011).

In addition, with the continuous emergence of PTMs types, there are various strategies for the detection, enrichment, identification and quantitative analysis of PTMs. Targeting a posttranslational modified protein is generally achieved by western blot, immunofluorescence, or immunohistochemistry, and Edman degradation, nuclear magnetic resonance (NMR), and mass spectrometry (MS) are used to identify the detailed sites of PTMs.Despite improvements in techniques for detecting and identifying PTMs, there are many limitations, such as the low abundance of enrichment of some post-translational modified proteins, which require more sensitive and resolution-based enrichment strategies and mass spectrometers. Several major hurdles remain in mass spectrometry-based clinical research. These include sample preparation, throughput, and complex data analysis. Proteins can be simultaneously modified by different PTMs at multiple sites. The combined action of multiple PTMs on the same or different proteins is called PTM crosstalk. A proteomic approach to detect PTM crosstalk was outlined by Mario et al. (Leutert et al., 2021). This further leads us to a profound understanding that PTMs do not exist in isolation, as we mentioned earlier that sulfide inhibits p65 NF-κB/STAT3 phosphorylation and acetylation to ameliorate diabetes-induced kidney injury. It has been found that different PTMs can also be regulated by regulating enzyme activity, and phosphorylation of certain E3 ligases can enhance or prevent ubiquitination of their protein targets (Hunter, 2007). Histone lysine crotonylation and histone acetylation have the same enzyme system, and the modified groups of the two are structurally similar, but they are functionally and mechanistically different (Tan et al., 2011). This evidence tells us that PTMs are interconnected and interact to control biological processes.

## Summaries and perspectives

The modulation of protein function through PTMs has become a pivotal regulatory mechanism in biological systems. This process serves as an interface between metabolism and physiological as well as pathological processes, thereby influencing the development of various human diseases. With the advancing research in this field, an expanding repertoire of HPTMs has been discovered, which are intricately linked to the pathogenesis of renal diseases.

This review article centers on the exploration of the significance of HPTMs, specifically acetylation, crotonylation, and Khib, as well as some non-HPTMs in renal diseases, particularly DKD and AKI. At the same time, through the discussion of protein glycosylation and phosphorylation in relation to kidney diseases, it is found that these two significant modifications are also involved in the development of kidney diseases, especially DKD. These modifications offer novel therapeutic prospects for the treatment of renal diseases. However, the link between novel PTMs and renal diseases remains unexplored and superficial. Since most of these modifications are reversible, it is probable that targeting the upstream or downstream intervention sites can pave the way for novel approaches in the treatment of renal diseases. This review highlights the regulatory role of acetyltransferases and de-acetyltransferases in diverse modifications, including p300, Khib, and lactylation, to control the enzyme activities of various substrates.

Many renal diseases involving lactylation have not been extensively studied with regard to HPTMs, except for H3K18la, which is enriched in ccRCC but not reported in lactylation in other renal diseases. The emerging field of lactylation in histones is currently under investigation in renal disease. The implementation of various omics technologies has led to the identification of novel types of PTMs and specific modification sites, which have improved and refined the modification profiles in renal diseases. Furthermore, since acyltransferases are not specific, we are investigating the possible relationship between lactylation and acetylation. This raises questions regarding the potential correlation between other modification and acetylation modification types, which should be investigated in future studies of the molecular mechanisms of renal diseases. The ongoing identification of novel types of HPTMs may open up new opportunities for the treatment of renal diseases.

It is interesting to explore the targets of novel PTMs in kidney disease. So far, we have learned that there are limited treatments for kidney disease, but the incidence and mortality of kidney disease are increasing. Epigenetic regulators control gene expression and can find more therapeutic targets by exploring the role and mechanism of different PTMs in kidney disease. However, more efforts are needed to apply these new ideas and ideas to the clinical field. Apabetalone was the first epigenetic modulator to conduct a phase 3 clinical trial in diabetic nephropathy with renal function as an endpoint. Therapeutic modulation can be performed directly by pharmacological modulators of specific enzymes involved and therapeutic use of desired substrates. But this remains a big challenge. Further exploration of the role PTMs play in the field of renal disease is still needed in the future, and given that interventions targeting epigenetic modifications in renal disease are still in clinical trials, it remains unknown whether early intervention in these pathways can treat the disease.
